# NBI and Laryngeal Papillomatosis: A Diagnostic Challenge: A Systematic Review

**DOI:** 10.3390/ijerph19148716

**Published:** 2022-07-18

**Authors:** Carmelo Saraniti, Salvatore Gallina, Barbara Verro

**Affiliations:** Division of Otorhinolaryngology, Department of Biomedicine, Neuroscience and Advanced Diagnostic, University of Palermo, 90127 Palermo, Italy; carmelo.saraniti@unipa.it (C.S.); salvatore.gallina@unipa.it (S.G.)

**Keywords:** papilloma, narrow-band imaging, larynx, laryngeal papillomatosis, diagnosis, differential diagnosis

## Abstract

**Highlights:**

▪ Laryngeal papillomatosis (LP) is considered a benign lesion, caused by HPV low-risk subtypes 6 and 11.
▪ Narrow-band imaging (NBI) endoscopy is a useful diagnostic tool for distinguishing between benign and malignant laryngeal lesions.
▪ Laryngeal squamous cell carcinoma (LSCC) and LP show the same type V pattern according to Ni classification on NBI.
▪ Epithelial surface and clinical features of LP should be researched to determine a correct differential diagnosis between LP and LSCC.

**Abstract:**

Narrow-band imaging (NBI) represents a valid aid in laryngeal squamous cell carcinoma (LSCC) diagnosis for detecting vascular changes. However, LSCC and laryngeal papillomatosis (LP) show similar vascular patterns that may lead to misdiagnosis and improper treatment. This review aims to deepen this NBI limit in order to stress a careful preoperative evaluation of laryngeal lesions. The research was carried out on PubMed, Web of Science and Scopus databases using specific keywords. The topic of research was assessed by these parameters: accuracy, sensitivity, specificity, and positive and negative predictive values. This review included only five articles: they demonstrated that NBI is better than white-light endoscopy in detecting LSCC and LP. They also reported that LP is frequently mistaken for LSCC, resulting in high rates of false positives using NBI. This is the first review that emphasized this NBI limitation in distinguishing between LP and LSCC in cases of a type V pattern of intraepithelial papillary capillary loop. Although NBI application increased the rate of early cancer detection, LP reduces NBI accuracy. This drawback may lead to misdiagnosis and improper treatment. Our advice is to be careful in cases of type V pattern on NBI and to research LP epithelial and clinical features because it could be a pitfall.

## 1. Introduction

Recurrent respiratory papillomatosis (RRP) is a benign and widespread respiratory disease among both adults and children. Its incidence is about 1.8 per 100,000 adults and 4.3 per 100,000 children [1]. It is caused by human papillomavirus (HPV) low-risk subtypes 6 and 11 [2]. Papilloma can occur anywhere in the aerodigestive tract, most often in the larynx, causing voice disorders and sometimes dyspnea due to airway obstruction. A recent study found that true vocal folds (TVFs) and anterior commissure (AC) are the most affected laryngeal sites [3]. The main challenge of RRP is its recurrent nature, which leads to several surgeries throughout life and in a few cases, more than four surgeries per year.

Since 2004, the introduction of narrow-band imaging (NBI) endoscopy has represented a valid aid in the diagnosis of laryngeal cancer: it is an optical technique that allows for better distinction between benign and malignant lesions than white-light endoscopy (WLE) [4]. NBI allows us to study the laryngeal mucosal and submucosal vascular patterns and to detect any vascular changes and/or neoangiogenesis, typical features of carcinoma. In 2011, Ni et al. proposed a classification of laryngeal lesions based on changes in intraepithelial papillary capillary loop (IPCL) patterns: Benign lesions have IPCL types I-III, malignant lesions have IPCL type Va,b,c, and precancerous or suspected malignant have IPCL type IV [5]. NBI use in clinical practice has achieved higher sensitivity and specificity for early laryngeal cancer than WLE [6]. However, this diagnostic tool has a main limitation: laryngeal papillomatosis (LP). Indeed, laryngeal squamous cell carcinoma (LSCC) and LP show the same IPCL changes: IPCL pattern type V according to Ni classification [7]. This issue may lead to misdiagnosis and so to over-treatment or, on the contrary, to undertreatment. 

Therefore, for the first time, this systematic review is aimed to deepen this NBI limit in order to highlight the importance of a proper and careful pre-operative diagnostic evaluation of laryngeal lesions for the best care of the patient.

## 2. Materials and Methods

### 2.1. Search Methodology

The bibliography available on PubMed, Scopus and Web of Science databases (up to 1 June 2022) was selected using the following keywords: “*Papillomatosis*” or “*Laryngeal papilloma*” and “*NBI*” or “*Narrow-Band Imaging*” (Table 1). First, the articles were selected by two independent authors (CS and BV) by reading titles and abstracts. Then, the included articles were read entirely to exclude those that did not meet the eligibility criteria in the study. Moreover, articles from references mentioned in previously selected articles were analyzed. The review was carried out according to PRISMA guidelines.

### 2.2. Eligibility Criteria

The inclusion criteria were: (1) studies about NBI use for laryngeal papilloma; (2) original articles; and (3) studies that reported at least one of accuracy (ACC), sensitivity (SE), specificity (SP) or positive or negative predictive value (PPV and NPV).

The exclusion criteria were: (1) studies about lesions involving sites other than the larynx and in particular the vocal cords; (2) editorials, reviews, conference abstracts, commentaries and theses; (3) studies that included fewer than 40 lesions; and (4) articles not written in English or Italian.

### 2.3. Data Analysis

The following data from included articles were reported: authors, year of publication, type of study design (retrospective or prospective), number of treated patients and/or lesions, IPCL type according to Ni classification, ACC, SE, SP, PPV and NPV. Data were collected on spreadsheets using Microsoft Excel (version 16.47.1). ACC is the ability of a diagnostic test to detect disease when it is present and to not detect it when disease is not present. SE represents the probability of positive test result in patients with disease. SP is the probability of negative test result in patients without disease. PPV is defined as the probability of having disease in patients with positive test results. NPV is the probability of not having disease in patients with positive test result [8].

## 3. Results

### 3.1. Study Selection

**Figure 1:**
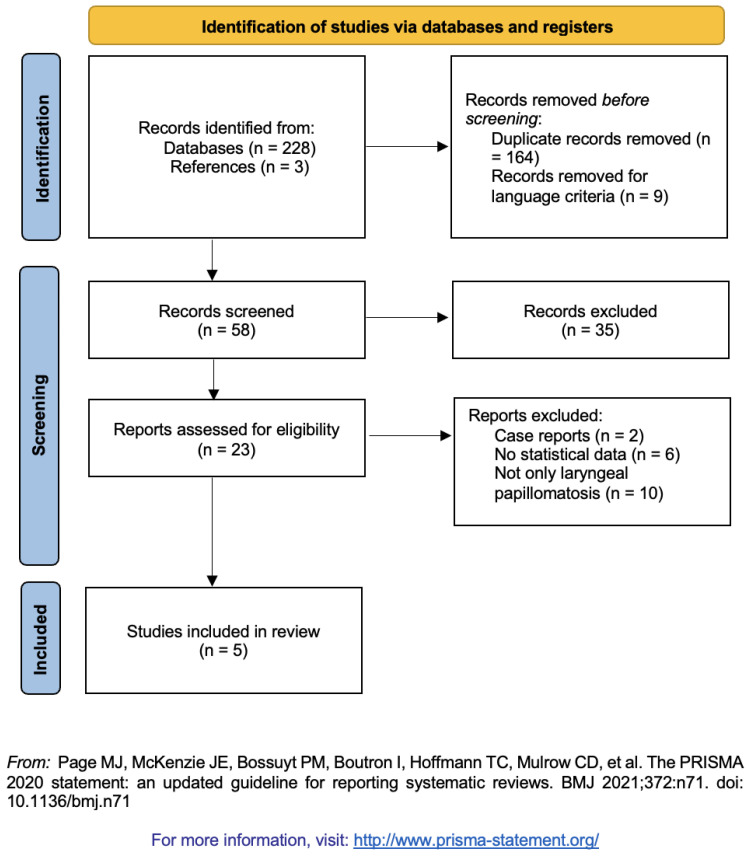
PRISMA 2020 Flow Diagram© of the study selection process from the literature search (from [9]).

Figure 1 shows the process of selection of the articles, using the Preferred Reporting Items for Systematic Reviews and Meta-Analyses (PRISMA) 2020 flow diagram [9]. Overall, 231 articles were selected from the systematic research in the databases and references. After removing the duplicates (164), 65 articles were analyzed, and 41 of them were excluded for reading titles and abstracts because of unrelated articles or language criteria. Then, 23 studies were read entirely by the two independent authors (CS and BV) and based on the eligibility criteria, only 5 articles were included in the present review (Table 2). 

### 3.2. Laryngeal Papillomatosis vs. Laryngeal Squamous Cell Carcinoma

Four of the five included studies reported measures of NBI diagnostic accuracy between LP and LSCC [7,10,12,13]. Studies were consistent to confirm high SE of NBI both for LP (92–94%) and LSCC (92–100%), better for the latter. Only in one study was SE for papillomatosis (41.67%) significantly lower than SE for LSCC (93.51%) [10]. Because of this, Lin et al. concluded that NBI is not suitable for diagnosing papillomatosis. On the contrary, NBI SP was better in the case of LP (92–100%) than in the case of LSCC (65–82%), showing another NBI limitation: white plaque that may cover the lesion and lead to false negative [6]. Only one study reported PPV and NPV data, 52.82% and 86.79% for LP and 91.14% and 68.18% for LSCC, respectively [10]. Particularly interesting is the analysis by Zwakenberg et al. [13], where they compared SE, SP, ACC, PPV and NPV between two groups: a group consisting of LP and LSCC together, the other group consisting of only LSCC. They demonstrated that papilloma increases the rate of false positives when using NBI, leading to misdiagnosis because both LP and LSCC have type V vascular patterns. In fact, as shown in Table 2, all included studies detected a type V pattern according to Ni classification for LP.

### 3.3. Laryngeal Papillomatosis: WLE vs. NBI

Tjon Pian Gi et al. carried out a study on 86 laryngeal lesions and compared SE and SP for laryngeal papillomatosis using WLE and NBI. They found that NBI increases sensitivity in detecting LP from 80% (with WLE) to 97% [11]. The study showed a low NBI SP (28%); however, as suggested by the authors, these data should not be considered because it would have been ethically incorrect to perform biopsies on healthy tissue.

## 4. Discussion

NBI endoscopy is usually defined as an “optical biopsy” because it allows for detecting early laryngeal cancer with higher sensitivity and specificity than WLE, almost like a real biopsy [4]. To engage the process, the operator simply presses a button to switch to NBI in the endoscope, and the larynx will appear cyan and green; NBI is based on narrow-band optical filters with two ranges of wavelength absorbed by hemoglobin: the range 400–430 nm (blue light) enhances the visualization of mucosal capillaries, and the range 525–555 nm (green light) allows for the observation of submucosal vessels [14]. Blood vessels of healthy vocal cords run parallel along the longitudinal axis of the vocal cords themselves, from anterior and posterior, and they do not form dots. In benign disease, IPCLs show larger and arborescent vessels but preserve a regular arrangement (IPCLs types I–II–III). In precancerous and suspected lesions, IPCLs usually appear as scattered and dark brown dots (IPCLs type IV). Malignant lesions are characterized by enlarged, brownish vessels, and IPCLs are destroyed with tortuous shape (IPCLs type V) [5]. Several studies demonstrated the high sensitivity and NPV of type V pattern for detecting early glottic cancer [15,16,17]; carcinogenesis is related to blood vessel changes and angiogenesis [18]. However, LP is also characterized by a type V pattern on NBI, and this may lead to misdiagnosis, as reported by all the studies included in this review. The most striking results were reported by Lin et al., who found sensitivity of 41.47% and 93.51%, respectively for papillomatosis and LSCC [10]. Moreover, Lin et al. [10] and Zwakenberg et al. [13] specified that LP showed type Va or Vb patterns, but no case of type Vc has been described. Unsurprisingly, NBI sensitivity in detecting LP was higher than WLE sensitivity (from 80% to 97%) [11]. 

Laryngeal papillomatosis is considered a benign lesion, usually caused by HPV low-risk subtypes 6 and 11 [2]. However, although cases are rare, LP may cause dysplasia and carcinoma: indeed, some studies, including that of Karatayli-Ozgursoy et al. [19], reported low rates of dysplasia (less than 20%) in RRP. This could be explained by the HPV low-risk subtypes in LP and HPV high-risk subtypes 16 and 18 in LSCC [20]. Moreover, Bolontrade et al. [21] studied the possible role of mouse blood vessel density in predicting the malignant progression of papillomata. They found that the angiogenesis occurs in the early stages of papilloma formation, reaching the conclusion that blood vessel density is not a predictor of malignant progression. HPV infection underlying RRP promotes neoangiogenesis and changes in the connective tissue [22]. Therefore, close attention must be paid to the preoperative evaluation of laryngeal lesions, always keeping in mind that NBI is a useful diagnostic tool but that it has a few limits: When otolaryngologists detect a type V pattern using NBI, they should distinguish between LP and LSCC, and, as suggested by many authors, they should seek other features of LP [12,13]. Despite the fact they used NBI endoscopy in combination with high-definition television (HDTV) that increased its diagnostic accuracy, Lukes et al. [7] highlighted the importance to study the epithelial surface and clinical features of LP. These main features of LP are symmetrical IPCLs, and papilloma appears as a pale and wart-like lesion, exophytic, with a raspberry-like surface and with capillaries along the central axis in each papilla [23]. Their procedure with the help of HDTV achieved similar sensitivity for LP (94%) and LSCC (100%) [7].

In 2015, the European Laryngological Society (ELS) classification of vocal fold vascular changes helps us to distinguish between LP and LSCC [24]. According to this new classification, vessels of the vocal folds should be studied in two steps: First assess the longitudinal vascular pattern and then assess the perpendicular vascular pattern. The former consists of vascular changes in only two dimensions of the vocal folds (width and length) and corresponds to benign lesions. The perpendicular vascular pattern describes the so-called IPCL: vessels that from the depth reach the surface of the vocal fold and then come back to the depth, drawing a loop. This latter pattern could be associated with both LP and LSCC. In this case, Arens et al. suggest paying special attention to the IPCL spiraling shape: LP has wide-angled turning points, whereas cancer lesions have narrow-angled turning points [6,24,25]. However, as stated by Sifrer et al., these vascular features are not easily visible: otolaryngologist experience and practice with high-quality equipment are necessary, fundamental, and in fact mandatory [11,14]. In 2010, the early days, Piazza et al. reported a high rate of false positives in the first six months of practice with NBI, defining the learning curve as the most crucial factor to outcome [26]. Moreover, intra-operative use of NBI significantly increases its accuracy, but the necessary equipment is not so widely present in the nose and throat (ENT) departments [14]. Indeed, the proper use of NBI is based on getting the tip of the endoscope as close as possible to the lesion to magnify the image and to provide higher diagnostic accuracy. 

Once a perfect image is achieved, it should be carefully and properly studied and assessed: NBI is a helpful tool in experienced hands.

### Study Limitations

The first and main limitation of this review is the tiny number of included studies: we had to exclude articles with few patients and/or without a clear distinction of parameters (SE, SP, ACC, PPV and/or NPV) between papilloma and other lesions of the vocal cords. Despite the differential diagnosis between LP and LSCC using NBI, it should be stressed that very few studies analyzed the rate of diagnostic error related to this misdiagnosis. Another drawback is related to the design study: A few studies were retrospective, but others did not include the type of design. Indeed, the retrospective nature of the study is inevitably produces results with less confidence and lower levels of evidence than prospective studies [27]. Moreover, all the included studies used Ni classification rather than ELS: this may have led to a higher percentage of false positive for carcinoma. It would have been interesting to compare the parameters (SE, SP, ACC and PPV and/or NPV) achieved using Ni or ELS classification.

## 5. Conclusions

To the best of our knowledge, this is the first review that has emphasized the limits of NBI: the difficulty distinguishing between laryngeal papillomatosis and cancer in the case of type V pattern of IPCL. Despite the introduction of this tool in the diagnostic routine that has significantly increased the rate of early detection of tumors, laryngeal papillomatosis seems to reduce NBI accuracy. This drawback may increase the rate of false positives, leading to misdiagnosis and thus to over- or undertreatment. However, only a few studies addressed this issue, and the high-quality equipment needed to overcome the obstacle is not available in all ENT departments. Therefore, our final advice is to be careful in case of type V pattern on NBI and to keep in mind that it could be a pitfall.

## Figures and Tables

**Table 1:** Number of articles based on keywords search strategy on databases.

Keywords	PubMed	Scopus	Web of Science
***Papillomatosis*** and ***NBI***	28	21	16
***Papillomatosis*** and ***Narrow-Band Imaging***	35	35	19
***Laryngeal papilloma*** and ***NBI***	13	13	8
***Laryngeal papilloma*** and ***Narrow-Band Imaging***	15	17	8

**Table 2:** Characteristics of the included studies.

Authors	Lukes P et al. [7]	Lin C et al. [10]	Tjon Pian Gi RE et al. [11]	Valls-Mateus M et al. [12]	Zwakenberg MA et al. [13]
**Year of publication**	2014	2021	2012	2018	2019
**Study design**	Not clear	Not clear	Prospective	Retrospective	Retrospective
**N° lesions**	109	123	86	41	178
**Ni classification [5]**	Type V	Types Va-Vb	Type V	Type V	Types Va-Vb
**Sensitivity**	94% (LP) 100% (LSCC)	41.67% (LP) 93.51% (LSCC)	97% (NBI) 80% (WLE)	/	92% (LP + LSCC) 92 (LSCC)
**Specificity**	100% (LP) 82% (LSCC)	92.93% (LP) 65.22% (LSCC)	28% (NBI) 32% (WLE)	/	68% (LP + LSCC) 88% (LSCC)
**Accuracy NBI**	/	/	/	95.32% (LP) 88.71% (LSCC)	77% (LP + LSCC) 84% (LSCC)
**Accuracy WLE**	/	/	/	82.92% (LP) 79.4% (LSCC)	/
**Positive predictive value**	/	52.82% (LP) 91.14% (LSCC)	/	/	61% (LP + LSCC) 73% (LSCC)
**Negative predictive value**	/	86.79% (LP) 68.18% (LSCC)	/	/	94% (LP + LSCC) 94% (LSCC)

Narrow-band imaging (NBI); white-light endoscopy (WLE); laryngeal papillomatosis (LP); laryngeal squamous cell carcinoma (LSCC).

## Data Availability

Publicly available datasets were analyzed in this study. These data can be found here: https://pubmed.ncbi.nlm.nih.gov/, https://www.webofscience.com/wos/woscc/basic-search, https://www.scopus.com; accessed on 1 June 2022.
